# Tuberculosis Diagnostic Methods: Clinical Applicability, Implementation Challenges, and Integrated Testing Strategies

**DOI:** 10.3390/pathogens15020142

**Published:** 2026-01-28

**Authors:** Eduarda Rabello, Fernanda de-Paris

**Affiliations:** 1 Specialization in Clinical Microbiology, Institute of Basic Health Sciences, Universidade Federal do Rio Grande do Sul, Porto Alegre 90035-903, RS, Brazil; 2 Laboratory Diagnostic Service, Microbiology Unit, Hospital de Clínicas de Porto Alegre, Porto Alegre 90035-903, RS, Brazil

**Keywords:** tuberculosis, diagnosis, diagnostic tests, specificity, sensitivity

## Abstract

Tuberculosis (TB) remains one of the leading causes of death from a single infectious agent worldwide, a burden further exacerbated by HIV co-infection and the increasing prevalence of drug-resistant strains. Although a wide range of laboratory diagnostic methods are currently available, their applicability, implementation, and clinical impact vary substantially across healthcare settings with different levels of complexity and resources. This review provides a comprehensive overview of the main laboratory diagnostic methods for active and latent TB, emphasizing their clinical applicability, implementation challenges, and role within integrated diagnostic strategies. Conventional approaches, such as smear microscopy and culture, are discussed alongside modern diagnostic technologies, including automated nucleic acid amplification tests (NAATs), loop-mediated isothermal amplification (LAMP), line probe assays (LPAs), next-generation sequencing (NGS), and lateral flow assays, highlighting their strengths and limitations in distinct epidemiological and operational contexts. Unlike existing WHO guidelines and prior reviews that predominantly focus on test performance and recommendation status, this review adopts an implementation-oriented perspective, critically examining diagnostic methods in light of real-world constraints, regional disparities, and evidence gaps. Particular attention is given to limitations related to laboratory infrastructure, biosafety, workforce capacity, and sustainability, as well as to under-addressed areas such as latent TB, metagenomic approaches, and the investigation of co-pathogens. By integrating WHO guidance with contextual and operational considerations, this review aims to support rational test selection and the development of flexible, integrated diagnostic workflows tailored to local health system capacity, patient populations, and clinical scenarios, thereby strengthening the effectiveness and equity of TB diagnostic strategies.

## 1. Introduction

In 2023, the World Health Organization (WHO) once again classified tuberculosis as the leading cause of death from a single infectious agent. It was estimated that 10.8 million people were infected with tuberculosis leading to 1.25 million deaths. Among the infected individuals, 6.1% were co-infected with the human immunodeficiency virus (HIV). In the same year, approximately 400,000 individuals with tuberculosis (TB) were estimated to have developed multidrug resistance or rifampicin resistance, and 44% of these cases received treatment. Among individuals with drug-susceptible tuberculosis, treatment demonstrated an efficacy rate of 88% [1]. Tuberculosis treatment consists of a combination of four drugs and requires a minimum duration of six months, which represents a challenge, as failure to adhere to the regimen may lead to the development of bacterial drug resistance. Currently, there are already multidrug-resistant (MDR) strains, defined as those resistant to the two most effective first-line drugs, isoniazid and rifampicin (RIF). In addition, extensively drug-resistant (XDR) strains exist, characterized by resistance to at least two second-line drugs [2,3].

Tuberculosis is typically diagnosed weeks or even months after the onset of infection, due to delayed or limited access to healthcare services and/or the presence of nonspecific symptoms, which increases the risk of transmission. Early diagnosis is crucial to enable effective treatment and, consequently, reduce new infections, as well as disease-related morbidity and mortality [3,4,5]. Early TB diagnosis is aligned with Sustainable Development Goal 3, established by the United Nations, which aims to ensure healthy lives by promoting access to healthcare and combating epidemics caused by various diseases, including tuberculosis, by 2030 [6].

The WHO, aiming to reduce tuberculosis cases and ultimately eradicate the disease, endorses and recommends the use of specific diagnostic methods for tuberculosis in its guidelines [2,7]. For a long time, the primary diagnostic methods for TB were smear microscopy and bacterial culture. Currently, molecular techniques, due to their higher sensitivity and specificity, have gained prominence in laboratories and received WHO support [7]. Moreover, they also exhibit a shorter turnaround time (TAT), defined as the total time between the test request and the delivery of the result, which is crucial for laboratory diagnosis, as it directly affects the promptness of clinical decision-making and the initiation of treatment [8].

Techniques such as automated nucleic acid amplification tests (NAATs), loop-mediated isothermal amplification (LAMP), lateral flow urine lipoarabinomannan assay (LF-LAM), line probe assay (LPA), next-generation sequencing (NGS), and interferon-gamma release assays (IGRAs) have been recommended by the WHO and represent promising alternatives [7].

In this context, this article aims to provide a comprehensive overview of the main laboratory and diagnostic methods for tuberculosis, emphasizing their applicability according to healthcare system complexity, clinical presentation, and epidemiological context, as well as their role within integrated diagnostic approaches. Unlike existing WHO guidelines and prior reviews, which primarily focus on test performance and recommendation status, this review critically examines tuberculosis diagnostic methods from an implementation-oriented perspective. By synthesizing WHO guidance with real-world constraints, evidence gaps, and contextual limitations across diverse healthcare settings, this article seeks to support rational test selection and the development of integrated diagnostic strategies beyond guideline-driven algorithms.

## 2. Search Strategy

This study is a narrative literature review with a descriptive and qualitative approach. A structured literature search was conducted primarily in PubMed, complemented by searches in journals accessed through institutional subscriptions and manual screening of reference lists. The search strategy combined English-language keywords related to tuberculosis diagnostics, including “tuberculosis”, “diagnosis”, “diagnostic tests”, “sensitivity” and “specificity”. The primary search focused on articles published between 2010 and 2025. However, seminal or historically relevant studies published before this period were also included when identified through reference list screening and deemed essential for contextualization or understanding of the evolution of tuberculosis diagnostic methods. Articles were considered eligible if they addressed diagnostic methods for tuberculosis and reported data on diagnostic performance or implementation-related outcomes, such as sensitivity, specificity, turnaround time, feasibility, or clinical applicability. Both original research articles and review papers were included. Exclusion criteria comprised editorials, letters, case reports, conference abstracts without full text, and studies not primarily focused on diagnostic performance. In addition, official guidelines, technical reports, and publicly available documents from recognized public health organizations were consulted to ensure a comprehensive overview of current diagnostic practices. Study selection was performed through title and abstract screening followed by full-text assessment when necessary, with emphasis on relevance to the scope of the review. Given the narrative nature of this review, no formal quantitative synthesis or standardized risk-of-bias assessment was performed; however, methodological limitations and potential sources of bias within the included studies were considered qualitatively during data interpretation. As this work is based exclusively on publicly available literature and does not involve human participants, ethical approval was not required.

## 3. Classic Methods

### 3.1. Bacterial Culture

Culture is considered the gold standard method for the diagnosis of tuberculosis and the detection of drug resistance, demonstrating high sensitivity and specificity [9,10]. This method allows the identification of different *Mycobacterium tuberculosis* species, even when bacterial cell counts are low [11]. Through culture, it is possible to multiply and isolate the mycobacteria from the inoculation of a clinical sample on specific media [12].

Classical culture methods use solid media, incubation in bacteriological incubators at temperatures between 35 °C and 37 °C, and visual colony reading. However, automated systems have also been developed, which utilize liquid culture media monitored by computerized systems capable of detecting cellular growth [12]. The BACTEC™ Mycobacterial Growth Indicator Tube 960 (MGIT) system is currently one of the most widely used. These systems exhibit higher sensitivity than solid media cultures and can be applied to most clinical samples [12,13,14]. In a retrospective study by Boldi et al. (2023) mycobacterial culture demonstrated a sensitivity of 98.8% and specificity of 100% for diagnosing pulmonary tuberculosis [15]. When comparing test sensitivity across different sample types, tracheal aspirates (97%) outperformed bronchoscopy samples (63.6%) [15].

Despite its many advantages, it is important to consider some challenges associated with the bacterial culture method. Strict biosafety practices are required, necessitating specialized equipment, trained personnel, and a biosafety level 3 laboratory due to the high infectious risk [15]. The bacterial growth time also poses a limitation for the early diagnosis of TB, as obtaining results can take between 14 days and 8 weeks [12].

Drug susceptibility testing (DST) is also performed using culture-based methods to determine whether *M. tuberculosis* isolates are susceptible or resistant to first-line and second-line antituberculosis drugs. DST may be carried out manually on solid media or through automated liquid systems. Among these, the BACTEC™ MGIT™ 960 system is widely employed because it enables both the detection of *M. tuberculosis* and the performance of DST. The system uses tubes containing predefined drug concentrations and a fluorescence-based oxygen sensor; as bacterial growth consumes oxygen, fluorescence increases and is continuously monitored and compared to a growth control, allowing the instrument to classify isolates as susceptible or resistant [12,16].

Although culture is considered the reference method for TB confirmation and drug susceptibility testing, it also presents relevant constraints. False negatives are frequently related to low bacillary burden, inadequate or poorly representative specimens, prior initiation of treatment, delays or inadequate storage and transport, excessive decontamination, and suboptimal incubation or culture conditions [17]. Conversely, false positives may arise from cross-contamination during specimen processing, mislabeling, sample mix-up, or incorrect identification of the bacteria. These constraints underscore the need for strict biosafety and quality-assurance procedures and for careful clinical–laboratory correlation when interpreting culture results [18,19,20].

### 3.2. Smear Microscopy

Smear microscopy is still a widely used technique for tuberculosis diagnosis; however, it does not allow for the identification of the mycobacterial species. High-complexity laboratories are not required to perform smear microscopy. It is a simple technique; however, it has low sensitivity, requiring a minimum concentration of 1000 CFU/mL in the clinical sample to be considered positive. Samples with low bacterial counts may yield negative results due to the difficulty of detecting bacilli [21]. Thus, collecting more than one sample per patient can improve the test’s sensitivity [22]. The method also shows low reproducibility, considering that sputum collection quality, slide preparation, staining, and microscopy procedures are critical factors that can directly affect the results [9].

This technique employs a special bacteriological staining method to identify acid-fast organisms, primarily mycobacteria. One of the most commonly used staining methods is Ziehl–Neelsen, in which bacteria present in the sample are stained with fuchsin [2,23]. Fuchsin is a dye that binds to the lipids in the bacterial cell wall, forming complexes that confer resistance to decolorization by acid-alcohol solutions. This characteristic gives mycobacteria their designation as acid-fast bacilli [12]. In the study by Singhal and Myneedu (2015), the Ziehl–Neelsen stain demonstrated a sensitivity of 22–43% [23]. Deng et al. (2021) reported similar sensitivity values, ranging from 20 to 30% [24].

When reading smear microscopy slides, at least one hundred fields must be examined, in which pulmonary cellular elements are observed (leukocytes, mucous fibers, and ciliated cells) [12]. Fluorescence microscopy using auramine stain shows approximately 10% higher sensitivity compared to conventional light microscopy. However, more accessible options exist, such as light-emitting diode fluorescence microscopy. This method uses long-lasting lamps, requires less energy, and has lower operational costs, offering greater sensitivity in resource-limited settings [25].

Smear microscopy is subject to important diagnostic limitations. False-negative results are frequent in paucibacillary disease, in children, people living with HIV, and extrapulmonary presentations, where the bacillary load is insufficient for microscopic detection. Test performance is also highly dependent on sample quality, staining technique, and reader expertise, which contributes to variability across settings [26,27,28]. Conversely, false-positive results may occur due to staining artefacts or the presence of nontuberculous mycobacteria, particularly in laboratories without rigorous quality control. These operational and biological factors reinforce that smear microscopy should not be used as a standalone diagnostic tool in clinically complex scenarios [29,30].

## 4. Latent Tuberculosis Diagnosis

Latent tuberculosis infection occurs when an individual is exposed to *M. tuberculosis*, resulting in a sustained immune response. In this condition, the individual remains asymptomatic, and no bacterial replication takes place [31,32]. When the immune system fails or the host becomes immunocompromised, the bacteria may reactivate and cause disease [33]. Approximately 5–15% of infected individuals progress to active tuberculosis, at which point the disease becomes contagious [31].

For the diagnosis of latent tuberculosis, the tuberculin skin test (TST), or Mantoux test, can be used. The test is based on a delayed-type hypersensitivity reaction that occurs after the intradermal injection, on the anterior surface of the forearm, of a purified protein derivative (PPD) from the mycobacteria. The PPD induces a local inflammatory response, and the maximum diameter of the resulting induration is measured 48 to 72 h after injection. However, this test has low specificity because the antigens present in PPD can also be found in other mycobacteria and in the Bacillus Calmette–Guérin (BCG) vaccine [11,34,35,36].

TST is less sensitive in immunocompromised patients, such as those using immunosuppressive agents and individuals infected with HIV. Due to these multiple factors that influence the test reaction, the cutoff value generally varies [37]. In 2022, three additional types of tuberculin were approved by the WHO, containing two *M. tuberculosis* specific proteins (ESAT-6 and CFP10). The Diaskin test, the C-TB skin test, and the C-TST offer higher specificity; however, none of them have been approved by regulatory authorities to date [4,34,38].

Other methods used for the diagnosis of latent infection include IGRAs. These tests incubate blood samples with *M. tuberculosis* specific antigens (ESAT-6 and CFP-10) and measures Interferon-Gamma (IFN-γ) production by lymphocytes sensitized to these antigens [11,39]. IFN-γ is quantified either by an enzyme immunoassay (ELISA) or by quantifying IFN-γ–secreting antigen-specific cells using an enzyme-linked immunospot assay (ELISpot) [40]. Compared with the tuberculin skin test, IGRA demonstrates greater accuracy and is not affected by the BCG vaccine [3,40,41]. Several IGRAs are currently commercially available, with the QuantiFERON-TB Gold Plus (Qiagen, Venlo, Netherlands) and the T-SPOT.TB (Oxford Immunotec, Oxfordshire, UK) assays being the most widely used [11].

The tuberculin skin test has the advantage of being less expensive, not requiring a laboratory environment, and being easier to use in screening settings [41]. On the other hand, IGRAs do not require a second visit for result reading and show fewer false-negative results in immunosuppressed individuals. In addition, they are specific for *M. tuberculosis* infection and therefore do not yield false-positive results in BCG-vaccinated individuals or those infected with nontuberculous mycobacteria (with the exception of *Mycobacterium kansasii*, *Mycobacterium szulgai*, *Mycobacterium marinum*, and *Mycobacterium riyadhense*, which contain the ESAT-6 antigen) [41,42].

There are 13 in vitro tests for the diagnosis of TB infection currently under development or being marketed, but not yet approved by the WHO. Of these, 12 are whole-blood IGRAs, and one of them—the GBTsol Latent TB Test Kit—uses a newly patented technology. In addition, five new skin tests and simplified versions of IGRAs based on lateral flow technology are in development. These tests are expected to offer higher specificity and to be suitable for use in peripheral healthcare units, thereby expediting TB diagnosis in these populations [37].

Both TST and IGRA are affected by important limitations related to false-positive and false-negative results. False-negative reactions are frequently associated with immunosuppression, recent infection, extremes of age, and operational errors in test administration or reading in the case of TST, or with delayed processing, inadequate incubation, or low mitogen response in IGRA [43,44]. Conversely, false-positive results may occur due to prior BCG vaccination or exposure to nontuberculous mycobacteria in TST, and, more rarely, due to cross-reactive mycobacterial species or borderline analytical variability in IGRA. These limitations underscore the need to interpret both tests in conjunction with clinical risk factors and epidemiological context [45,46].

## 5. Lateral Flow Assays

In 2019, the Alere Determine TB-LAM Ag lateral flow assay, performed on urine samples, was recommended by the WHO to assist in the diagnosis of active tuberculosis in individuals infected with HIV [47]. Therefore, the Alere Determine TB-LAM Ag assay is performed manually by applying 60 µL of urine to the test strip and incubating it at room temperature for 25 min. The result is visualized through the presence of bands on the strip—one test band and one control band—whose intensity is compared with the band intensities on a reference scale provided by the manufacturer [7].

Lipoarabinomannan (LAM) is a glycolipid present in the cell wall of *M. tuberculosis* and can be used as a biomarker for TB diagnosis [48]. During blood filtration in the kidneys, glomerular endothelial cells form a network with pore sizes sufficient to allow the passage of membrane-derived molecules from *M. tuberculosis* or extracellular vesicles carrying LAM, which are subsequently excreted in the urine [49]. This type of test demonstrates higher sensitivity and specificity in HIV-positive individuals, particularly those with CD4 cell counts below 100 cells/µL, and is therefore not recommended for other groups [32].

Recently, another test was developed with the aim of improving the sensitivity while maintaining the specificity of the Alere Determine TB-LAM Ag assay. The Fujifilm SILVAMP TB LAM test (FujiLAM) employs a pair of high-affinity monoclonal antibodies directed against the 5-methylthio-D-xylopyranose epitope, which is specific to *M. tuberculosis*, and incorporates a silver amplification step that enhances the visibility of the test and control lines on the lateral flow assay. This approach enables the detection of LAM concentrations approximately 30 times lower than those detectable with the Alere Determine TB-LAM Ag test in urine samples. FujiLAM has demonstrated approximately 30% higher sensitivity in HIV-positive patients compared with the Alere Determine TB-LAM Ag assay, while maintaining a specificity of 95.7% [50,51,52].

The diagnostic accuracy of urine LAM assays is strongly dependent on pre-analytical and analytical factors that may lead to false-negative or false-positive results. False negatives are commonly associated with low systemic bacillary burden, localized or paucibacillary disease, preserved immune function in people living with HIV, inadequate sample volume or dilution, and degradation of the antigen due to suboptimal storage or handling [53,54]. Conversely, false-positive reactions may result from cross-reactivity with nontuberculous mycobacteria, renal disease–related proteinuria, bacterial coinfections, or subjective interpretation of weak test bands, particularly when operational conditions are not strictly controlled [53,55]. These limitations emphasize the need to interpret LAM results within the clinical and epidemiological context and, when available, in combination with complementary diagnostic methods [56].

## 6. Molecular Methods

Molecular methods provide several advantages in the laboratory diagnosis of tuberculosis, particularly regarding the rapid turnaround time, test standardization, and reduced biosafety requirements compared with culture-based methods using liquid or solid media.

The WHO has supported the expanded use of rapid molecular tests to detect both tuberculosis and antimicrobial resistance as part of a strategy to improve diagnostic accessibility and ensure more comprehensive and equitable care for individuals with TB. This is because early and accurate detection of the disease enables prompt initiation of appropriate treatment, contributing to more effective tuberculosis control [12].

### 6.1. Automated Nucleic Acid Amplification Tests (NAAT)

These methods detect TB and mutations associated with resistance to first-line antimicrobials, such as rifampicin and isoniazid, as well as to second-line agents, including fluoroquinolones, ethionamide, and amikacin, yielding results within a few hours. The efficiency of these approaches is particularly valuable in settings where large numbers of tests are performed daily and offers a suitable alternative for laboratories with limited resources [13,57,58].

Polymerase Chain Reaction (PCR) is currently the most widely used technique and can be performed using automated platforms such as Xpert^®^ MTB/RIF, Xpert^®^ MTB/RIF Ultra (Cepheid, Sunnyvale, CA, USA), and Truenat MTB (Molbio, Goa, India) [59]. PCR enables the amplification of specific DNA segments millions of times through the use of primers. This high amplification capacity confers excellent sensitivity, allowing the detection of very small amounts of genetic material [60,61].

The Xpert^®^ MTB/RIF assay, developed by Cepheid Innovations (USA), represented a major advance in the diagnosis of tuberculosis and in the detection of rifampicin resistance, as it is a simple and rapid test capable of delivering results in up to two hours [9,62]. This assay is an automated, real-time, semi-quantitative PCR used for the simultaneous detection of the M. tuberculosis complex and its resistance profile to RIF. It amplifies the rifampicin resistance–determining region of the rpoB gene in clinical specimens [13,63]. Once the sample is loaded into the cartridge, all testing steps—from amplification to PCR-based detection—are performed automatically within the enclosed cartridge system, thereby minimizing the risk of contamination [64].

An improved version, the Xpert^®^ MTB/RIF Ultra, is also available and offers greater sensitivity and improved performance for TB detection in patients with HIV infection [65,66]. It is used both to detect *M. tuberculosis* DNA and to assess patient infectiousness based on its semi-quantitative output [15,67]. As with previous generations, the Ultra detects RIF resistance by employing four probes targeting the rpoB gene. Compared with earlier versions, Ultra test cartridges contain a larger DNA amplification chamber and incorporate two multi-copy amplification targets for TB (IS6110 and IS1081) [63], resulting in a lower limit of detection of 16 CFU/mL. These modifications increased the overall sensitivity of the Ultra from 85% to 88% [7,59,68], and this assay is particularly recommended for paucibacillary forms of TB, such as TB of the central nervous system [32,69].

Xpert^®^ MTB/RIF and Xpert^®^ MTB/RIF Ultra are useful in paucibacillary tuberculosis due to their ability to provide semiquantitative results derived from cycle threshold values. These assays classify *M. tuberculosis* concentrations into “very low,” “low,” “medium,” or “high” categories, allowing the identification of disease states characterized by low bacillary burden. The “very low” and “low” semiquantitative categories are particularly relevant for paucibacillary presentations, which are frequently observed in paediatric tuberculosis, where conventional methods such as smear microscopy have limited sensitivity [70,71]. This semiquantitative output enhances the clinical interpretability of molecular results in populations with low bacterial loads [72].

The Xpert MTB/XDR assay was designed to simultaneously detect mutations associated with resistance to multiple first and second-line anti-tuberculosis drugs, particularly in cases of extensive drug resistance [59,73]. This test was redesigned to improve mutation coverage for isoniazid, differentiate between low and high-level resistance to isoniazid and fluoroquinolones, identify resistance to ethionamide, and distinguish cross-resistance from individual resistance to second-line injectable agents. The analysis time was reduced to 90 min, and the assay’s sensitivity was also enhanced [74].

Other real-time PCR assays include Truenat MTB, MTB Plus, and MTB-RIF Dx, developed by Molbio (India). MTB and MTB Plus are used as initial diagnostic tests for TB detection, whereas MTB-RIF Dx is employed for the detection of rifampicin resistance. The analysis is performed on automated, battery-powered devices that extract, amplify, and identify specific gene targets, enabling the rapid diagnosis of TB infections. These devices are simple to operate and can be used in peripheral laboratories with minimal infrastructure, providing results in under one hour [7].

Although WHO recommends Xpert MTB/RIF as the initial diagnostic test, the underlying evidence is largely derived from studies in controlled settings and adult pulmonary TB. While rapid molecular tests demonstrate high overall sensitivity and specificity, their performance is not uniform across clinical contexts. False negative results may occur and are mainly associated with low bacillary burden, insufficient or poorly collected specimens, the presence of inhibitory substances, inadequate storage or transport, or incomplete lysis and DNA extraction due to incorrect sample–reagent handling [75,76]. Conversely, false positives may result from cross-contamination during collection or processing, detection of residual non-viable mycobacterial DNA in previously treated patients, or from mutations of uncertain significance and mixed bacterial populations affecting rifampicin-resistance calls. These factors highlight the need for strict sample-handling procedures and careful clinical interpretation of Xpert results [77].

### 6.2. Loop-Mediated Isothermal Amplification (LAMP)

It is based on DNA amplification at a single, constant temperature (isothermal), eliminating the need for a thermocycler. In this assay, multiple DNA strands can be accurately amplified repeatedly within approximately one hour [24,78]. In the LAMP technique, primers bind to the target region of the DNA sequence and drive the amplification process. Another essential component of this method is the DNA polymerase enzyme, which unwinds the DNA strand under isothermal conditions, enabling amplification [60,79]. The current application of LAMP for tuberculosis relies on the amplification of the gyrB and IS6110 target genes of the *Mycobacterium tuberculosis* complex [80].

The results can be visualized by different methods, such as a visible color change to the naked eye, fluorescence under ultraviolet light, or turbidity formation, depending on the dye or system used [24]. Because it is a simple and cost-effective method that does not require sophisticated equipment, it can be widely applied, especially in smaller laboratories with limited resources [24,59,81]. Conversely, the major disadvantage of LAMP is that the use of multiple primers can lead to nonspecific amplifications, resulting in false-positive results [82]. In the study by Deng et al. (2021), the sensitivity and specificity of LAMP for detecting *M. tuberculosis* in bronchoalveolar lavage fluid were 73% and 99%, respectively [24].

A commercial assay based on the LAMP technique is the Loopamp™ (TB-LAMP) kit, developed by Eiken Chemical Company. This assay is manual and takes less than one hour to perform, and its results can be read with the naked eye under UV light. It is a rapid diagnostic test that does not require sophisticated infrastructure and may eventually be considered an alternative to smear microscopy [7].

The diagnostic accuracy of the LAMP assay is influenced by several factors. False-negative results are more likely in samples with very low bacillary load or in extrapulmonary disease, where validation remains limited [83,84]. On the other hand, the use of multiple primers increases the risk of nonspecific amplification, which may lead to false-positive results. Because LAMP remains largely a manual, multi-step procedure, operational variability during specimen handling, reaction setup, and result interpretation may increase the risk of analytical errors, potentially leading to false-negative or false-positive results. These factors highlight the need for rigorous workflow separation, quality-control procedures, and cautious clinical interpretation of LAMP results. For this reason, LAMP is best applied as a complementary screening tool rather than a definitive diagnostic test in all scenarios [85,86].

### 6.3. Line Probe Hybridization (LPA)

The LPA is based on reverse-hybridization DNA strip technology and detects DNA from the *M. tuberculosis* complex, allowing the determination of the bacterial strain within the complex and the antimicrobial resistance profile [9]. This occurs through the binding of amplified DNA products from these bacteria to probes that target specific regions of the *M. tuberculosis* genome, common mutations associated with drug resistance, or the corresponding wild-type DNA sequence [87]. These assays are more complex compared with the Xpert test, for example, yet they are capable of detecting resistance to several first and second-line antimicrobials by identifying genetic mutations in common variants [9,59]. Results can be obtained within 5 h [7].

This technique involves three steps: DNA extraction from clinical samples or cultured isolates, multiplex PCR amplification, and reverse hybridization. Finally, it is possible to observe the binding of the amplicon to mutation probes and wild-type probes, or the absence of such binding [87]. Some steps can be automated, making the assay faster and reducing the risk of contamination [7].

First-line LPAs are designed to detect tuberculosis and resistance to rifampicin and isoniazid in respiratory samples. One assay that is widely used today is the GenoType^®^ MTBDRplus, which has two versions [88,89]. These assays include rpoB probes to detect rifampicin resistance, katG probes to detect mutations associated with high-level isoniazid resistance, and inhA promoter probes to detect mutations generally associated with low-level isoniazid resistance [7].

The GenoType MTBDRsl is an assay designed to detect mutations associated with fluoroquinolones and second-line injectable drugs. The first version detects mutations in the quinolone resistance–determining region of gyrA and rrs. The second version additionally detects mutations in the gyrB region and in the eis promoter. Both the first and second-line assays rely on the same principle: the bands observed correspond to either a wild-type or a resistance probe and can be used to determine the drug susceptibility profile of the analyzed sample [7].

The study by Kanade et al. (2023) compared the performance of the Xpert^®^ MTB/RIF assay and the LPA in detecting *M. tuberculosis* and antimicrobial resistance [9]. Both tests were evaluated against the mycobacterial growth indicator tube (MGIT) 960 liquid culture system and drug susceptibility testing. The Xpert^®^ MTB/RIF assay demonstrated a sensitivity of 92.1%, while the LPA showed a sensitivity of 90% [9].

LPAs provide rapid genotypic information on drug resistance but are subject to important biological and methodological limitations. False-negative resistance results may occur in the presence of low bacillary load, poorly collected or degraded specimens, inhibitory substances, insufficient DNA extraction, resistance-conferring mutations located outside the targeted genomic regions, or heteroresistance below the assay’s detection threshold [90,91,92]. Conversely, apparent false-positive resistance may arise from cross-contamination or amplicon carry-over, detection of polymorphisms or mutations of uncertain clinical significance, mixed bacterial populations, and subjective interpretation of weak or borderline bands. These limitations underscore the need for strict quality-control procedures and careful clinical–laboratory correlation, positioning LPA as a complementary tool within broader diagnostic and drug-resistance testing strategies rather than a standalone assay [91,92,93].

### 6.4. Next-Generation Sequencing (NGS)

This method detects resistance to a larger number of antimicrobials compared with other tests, including those used in more modern treatment regimens. By amplifying selected genes, it can identify specific resistance-associated mutations with greater accuracy [58,59].

Metagenomic next-generation sequencing (mNGS) directly extracts fragments of DNA or RNA from clinical samples without isolating specific pathogens and performs sequencing independently. These sequences are then compared with databases encompassing known pathogenic microorganisms, thereby enabling the detection of *M. tuberculosis*. In this way, mNGS also demonstrates potential for identifying coinfections and/or determining antimicrobial resistance [65,94,95].

With technological advancements, the turnaround time has been drastically reduced, allowing results to be obtained within 24 h [96]. In extrapulmonary tuberculosis, the pathogen may invade other organs such as the brain, bones, and joints; therefore, corresponding samples can be used for diagnosis. Studies employing mNGS for the diagnosis of tuberculous meningitis have demonstrated excellent performance, with a detection rate of 95.65% using cerebrospinal fluid samples [65,97].

Although mNGS demonstrates high sensitivity and specificity, it does not offer cost–benefit advantages when compared with the average cost of Xpert. Sequencing results depend on the concentration of target sequences in the sample; therefore, both the cost and the analysis time increase with sequencing depth [65,98,99]. The use of this assay has been limited in resource-constrained settings due to the high investment costs and the need for specialized technical expertise and bioinformatics support for its implementation [58,100,101].

Targeted next-generation sequencing (tNGS) amplifies and sequences a selected set of genes or genomic regions that are associated with a specific pathogen or phenotype, such as drug resistance, making it useful in cases with low pathogen load [65,102]. Sequencing results can be obtained within a few hours, while the overall turnaround time from primary sample to final report ranges from 1 to 10 days. In addition to sputum, other sample types—such as stool and cerebrospinal fluid—can also be used [58].

The customization of tNGS assays allows them to be applied in diverse contexts. Assays can be selected based on available sequencing capabilities, adapted to the required throughput, and target genes can be updated according to new evidence or context-specific differences, with minimal changes needed to existing infrastructure and testing procedures [58]. The tNGS method requires custom primers for specific pathogens, which facilitates the creation of tailored genetic panels [103]. tNGS can serve as a rapid and complementary diagnostic tool in the context of drug-resistant tuberculosis treatment regimens. Portable NGS devices with reduced costs are currently being developed, which may increase the accessibility of this technology [58,104].

Some tests that detect and identify mycobacterial species and antibiotic resistance based on targeted NGS include the Deeplex^®^ Myc-TB assay, developed by Genoscreen (Lille, France); the AmPORE-TB^®^ assay, developed by Oxford Nanopore Diagnostics (Oxford, UK); and the TBseq^®^ assay, developed by Hangzhou ShengTing Medical Technology Co. (Hangzhou, China) [7].

At present, routine implementation is mainly restricted to reference laboratories and national surveillance programs, where tNGS is used to complement or replace culture-based drug susceptibility testing, particularly in cases with suspected drug resistance or inconclusive molecular results [58,105,106]. In contrast, mNGS currently represents a technology with important future potential rather than routine clinical use. mNGS enables hypothesis-free pathogen detection directly from clinical specimens and may be especially useful for paucibacillary or extrapulmonary TB and for the investigation of differential diagnoses or co-infections [107,108,109]. As a result, mNGS is presently applied mostly in research contexts or selected complex clinical cases, while further technical optimization, cost reduction, and validation studies will be required before widespread adoption in TB diagnostic workflows can be achieved [108,109].

Although NGS offers expanded coverage of resistance-associated mutations and the potential for comprehensive genomic characterization, its clinical implementation is still constrained by important limitations. Routine use requires specialized bioinformatics pipelines, trained personnel, and high-complexity laboratory infrastructure, which restricts availability in resource-limited settings [110,111]. In addition, the risk of cross-sample or environmental contamination during nucleic acid extraction and library preparation may compromise result reliability, particularly in laboratories without strict quality assurance procedures [111,112].

The diagnostic yield of NGS is also reduced in paucibacillary specimens, in which the low concentration of mycobacterial DNA limits sequencing depth and mutation detection [113,114]. Furthermore, the clinical interpretation of genomic variants—especially those of uncertain significance—remains challenging and often requires expert consultation. Taken together, these factors indicate that, while NGS holds substantial future potential for TB diagnosis and resistance surveillance, its current applicability in routine workflows is better suited to reference laboratories and confirmatory testing rather than universal frontline use [111]. Therefore, current applications of NGS in TB diagnostics should be viewed as complementary to rapid molecular and culture-based methods, with progressive expansion anticipated as costs decrease, bioinformatics workflows become more standardized, and technical capacity increases.

NGS expands the detection of resistance-associated mutations and provides comprehensive genomic information; however, its diagnostic performance varies across clinical and laboratory contexts. False-negative results are mainly associated with low mycobacterial DNA content due to low bacillary burden, poorly representative or degraded specimens, predominance of host DNA, incomplete extraction, limited sequencing depth, resistance-conferring mutations in poorly covered or untargeted regions, or heteroresistance below the detection threshold [109,115]. On the other hand, false-positive finding—s may arise from cross-contamination or amplicon carry-over during library preparation, sequencing or PCR artefacts, mixed bacterial populations, and misclassification of polymorphisms or variants of uncertain clinical significance as resistance. Together, these constraints underscore the need for stringent laboratory and bioinformatics quality-control procedures and careful clinical interpretation, positioning NGS primarily as a complementary or confirmatory tool in reference settings rather than a universal frontline diagnostic method [58,116].

## 7. Emerging Diagnostic Methodologies

In addition to conventional diagnostic tools (such as smear microscopy, culture, and molecular assays), several emerging methodologies have shown potential to optimize tuberculosis diagnosis in the future. Recent studies have explored host-response biomarkers identified through proteomic and metabolomic analysis in blood, sputum, and other biological samples, which may contribute to distinguishing active disease from latent infection and to monitoring treatment response beyond current approaches [117,118,119]. Likewise, metabolomics combined with machine-learning techniques has been investigated to detect metabolic signatures associated with pulmonary tuberculosis, with promising diagnostic performance [120].

Artificial intelligence (AI)-assisted analysis of radiologic imaging, including chest radiography and computed tomography, is also being developed to support automated detection and triage of TB-related abnormalities, in some cases achieving accuracy comparable to expert human interpretation [121,122]. Furthermore, the field of nano-enabled diagnostic technologies, such as nanoparticle-based biosensors and nanostructured detection platforms, has proposed rapid and highly sensitive methods capable of identifying mycobacterial components or host biomarkers at very low concentrations, potentially enabling portable and low-cost testing. Although most of these approaches still require extensive clinical validation, standardization, and assessment of feasibility in real-world settings, they represent promising complementary pathways for the future expansion of the TB diagnosis [123,124].

## 8. Practical Implications for the Clinical Laboratory

Given the current epidemiological landscape of tuberculosis, characterized by high morbidity and mortality rates, the implementation of appropriate, rapid, and sensitive diagnostic methods is essential for disease control. The selection of the most suitable diagnostic tests should be based on multiple factors, including the complexity of the healthcare service, laboratory infrastructure, workload, available financial resources, the epidemiological profile of the population served, and the clinical or epidemiological purpose of the diagnosis, which directly affects the required turnaround time to meet the needs of the target population. In this context, Table 1 provides a comparative overview of the diagnostic tests addressed in this review, highlighting their clinical use, turnaround time, sample requirements, costs, biosafety level and major limitations across different patient populations and laboratory settings.

In high-complexity hospitals with infectious disease units, emergency departments, intensive care units, and inpatient care for patients with severe forms of TB, the adoption of automated molecular methods, such as Xpert MTB/RIF Ultra or Xpert MTB/XDR, is recommended. These assays enable rapid detection of TB and resistance to essential antimicrobials with a high degree of sensitivity. They have a reduced turnaround time, with results available in less than two hours, making them ideal for immediate clinical decision-making, such as in emergency care settings. Additionally, these facilities can also perform LPAs, primarily as confirmatory tools following initial molecular detection of resistance. Although these tests require greater technical expertise, they provide detailed genotypic information that is valuable for guiding therapeutic decisions.

In medium-complexity hospitals or regional laboratories with moderate infrastructure, moderately complex NAATs, such as Xpert MTB/RIF Ultra or Xpert MTB/XDR, can also be utilized. These assays provide higher analytical throughput and are suitable for laboratories managing a moderate volume of samples. Additionally, they can be combined with bacterial culture and susceptibility testing to monitor resistance to second-line antimicrobials and to complement the diagnosis in more complex cases.

In low-complexity hospitals, particularly those located in remote areas or facing shortages of human and financial resources, methods such as smear microscopy can still be employed as a screening tool, although their low sensitivity limits their effectiveness. These settings would also benefit from the implementation of LAMP, due to its simplicity and superior performance, providing results within one hour with minimal equipment. Other alternatives include simplified versions of molecular assays currently under development, designed to overcome barriers related to cost and infrastructure.

In emergency departments, urgent care units, or infectious disease wards that admit patients previously diagnosed with HIV, lateral flow assays such as Alere Determine TB-LAM Ag and FujiLAM can be useful alternatives for rapid TB diagnosis. Additionally, individuals who have been in contact with infected people or who are at high risk of infection, such as immunocompromised patients, may undergo latent tuberculosis testing using assays for example, the QuantiFERON-TB Gold-Plus IGRA.

For laboratories processing a high volume of samples or specimens originating from remote areas, standardization of workflows and automation of laboratory processes are essential strategies. In these contexts, automated NAATs with batch processing capability, combined with liquid culture systems (BACTEC MGIT 960), ensure traceability, quality control, and high sensitivity. Additionally, tNGS-based assays can be employed for resistance monitoring and molecular surveillance, particularly in reference centers, due to their high accuracy and ability to detect multiple genetic markers in a single assay.

For epidemiological studies and laboratory surveillance, such as those conducted in research institutes or national reference centers, it is essential to implement methods that enable not only diagnosis, but also molecular typing and identification of mutations associated with drug resistance. In this context, techniques such as LPA and NGS stand out for providing robust data to guide public health policies and strategies for controlling drug-resistant TB. However, due to their high costs and infrastructure requirements, these tests should be centralized in national reference laboratories with efficient sample workflows and adequate bioinformatics support.

The diagnosis of latent tuberculosis infection (LTBI) is also relevant as it is part of the investigation of active TB, in which screening, clinical assessment, and preventive strategies are inter-related. TST and IGRA can be used in primary care, outpatient clinics, and TB-control program settings, where they support screening of contacts and high-risk groups. In programmatic contexts, LTBI testing is mainly used as a screening tool in individuals at increased risk of infection or progression—such as household and close contacts of bacteriologically confirmed cases, people living with HIV, candidates for immunosuppressive therapy, healthcare workers, and children exposed to infectious adults. In all of these groups, testing must be preceded by clinical evaluation.

The clinical value of LTBI diagnosis is that it informs decisions about whether preventive treatment should be started and how it should be managed. These decisions are not based only on TST or IGRA results, but also on the individual risk of progression to active disease, which depends on factors such as immune status, age, recency and intensity of exposure, comorbidities, and the epidemiological context. People at very high risk—including those living with HIV, recent contacts, and patients starting biologic or other strong immunosuppressive therapies—may be prioritized for preventive treatment even when some diagnostic uncertainty remains, especially in high-burden settings.

Occupational TB screening among laboratory professionals who handle potentially infectious specimens is also important and relies on structured protocols that combine periodic clinical and occupational assessments with targeted diagnostic testing to identify both latent TB infection and active disease. Routine screening includes symptom-based clinical evaluation, the use of TST or IGRAs to detect prior exposure or recent infection, and chest radiography to exclude active pulmonary TB when indicated. Conversion of TST or IGRA results is a key marker of recent infection and prompts further investigation and consideration of preventive therapy. In cases with clinical or radiological suspicion of active disease, microbiological testing of respiratory samples—such as smear microscopy, rapid molecular assays, and mycobacterial culture—can be performed to confirm diagnosis and assess drug susceptibility. The frequency of screening is determined by occupational risk and institutional guidelines, with the main objective of early detection, timely treatment initiation, and prevention of transmission within laboratory settings and the wider community.

It is also essential for each facility to establish an integrated diagnostic workflow, encompassing proper sample collection and transportation through to the delivery of results. This workflow should consider the use of rapid screening tests, followed by confirmation with culture and susceptibility testing, and, in selected cases, the application of advanced molecular tools. Another important consideration is the heterogeneity in test performance and the occurrence of false-positive and false-negative results. No single method is sufficient for all clinical scenarios; therefore, integrated diagnostic workflows are essential to mitigate individual test limitations and support context-specific decision-making. Well-defined strategies allow for resource optimization, increased diagnostic coverage, and reduced turnaround time, the interval between clinical suspicion and treatment initiation.

It is important to note that the diagnosis of extrapulmonary and pediatric tuberculosis is particularly challenging due to the paucibacillary nature of disease and the difficulty in obtaining appropriate clinical specimens, which compromises the performance of available diagnostic tests [125]. In children, microbiological confirmation remains uncommon due to low bacillary burden and limited ability to obtain expectorated sputum, resulting in reduced sensitivity of smear microscopy and culture [126]. Molecular assays such as Xpert MTB/RIF and Xpert MTB/RIF Ultra represent important advances, offering higher sensitivity and rapid results, particularly when applied to multiple or alternative specimen types, including gastric aspirates, induced sputum, nasopharyngeal samples, stool, and urine. However, even these tests fail to detect a substantial proportion of cases, reinforcing that negative results cannot reliably exclude disease [127,128].

For extrapulmonary TB, similar limitations are observed, as non-respiratory specimens such as cerebrospinal fluid, pleural fluid, lymph node aspirates, or tissue biopsies often contain low bacillary loads, leading to variable diagnostic yield. Consequently, current evidence supports the use of sensitive molecular tests as part of an integrated diagnostic approach, combined with clinical, radiological, and histopathological findings, rather than reliance on a single assay [7].

Despite major advances in tuberculosis diagnostics and the detailed guidance issued by the WHO, important limitations affect the applicability, implementation, and real-world clinical impact of recommended diagnostic algorithms across diverse health-care settings. Regional disparities in infrastructure, epidemiology, and health system capacity further complicate the universal application of WHO diagnostic guidance. Many high-burden countries face constraints related to laboratory infrastructure, biosafety requirements, supply chain stability, and trained personnel, which may limit the feasibility and sustainability of implementing high-complexity molecular or sequencing-based technologies. These limitations highlight that WHO diagnostic guidelines, while essential, should not be interpreted as universally prescriptive. Instead, they should be integrated into flexible, context-sensitive diagnostic workflows that account for local epidemiology, health system capacity, and patient populations.

Regarding diagnostic tests the WHO guidelines do not address the integration of metagenomic approaches or the investigation of co-pathogens, despite the broader technological capacity of these methods in other clinical contexts, representing a potential area for future expansion of diagnostic guidelines as mentioned in this review. In addition, the WHO module focuses on the diagnosis of active tuberculosis and does not address latent TB infection, which is covered in this review and represents an important complementary dimension of tuberculosis diagnostic strategies.

Finally, it is essential that healthcare managers and professionals be trained to understand the different available methods, their advantages, and limitations, in order to implement appropriate strategies. Therefore, there is no single test that is ideal for all settings. The selection of the most suitable diagnostic method should be carried out strategically and contextually, ensuring accessibility, accuracy, rapidity, and a positive impact on clinical management and TB control. Rather than reiterating isolated test recommendations, this review emphasizes integrated diagnostic approaches that combine molecular, culture-based, and adjunctive assays according to clinical suspicion, bacillary burden, and service complexity. The establishment of integrated laboratory networks, with well-defined workflows and continuous technical support, is crucial for the effectiveness of tuberculosis control programs.

## Figures and Tables

**Table 1:** Characteristics and practical limitations of tuberculosis diagnostic methods.

Diagnostic Test	Sample Type(s)	TAT	Cost	Biosafety Level	Clinical Use	Limitations
Smear microscopy	Sputum, induced sputum, gastric aspirate, tracheal aspirate, BAL, lymph node aspirate, tissue fragments, CSF, cavity fluids, urine	~1–2 h	Very low	BSL-2	Initial screening; detection of highly bacillary pulmonary TB	Low sensitivity in HIV+, children and paucibacillary disease; limited value in extrapulmonary TB; does not detect species or resistance; operator- and sample-dependent performance; moderate biosafety needs during slide preparation
Bacterial culture	Sputum, induced sputum, gastric aspirate, tracheal aspirate, BAL, lymph node aspirate, tissue fragments, CSF, cavity fluids, urine, blood/bone marrow	~14 days–18 weeks	Medium to high	BSL-3	Diagnostic confirmation (“gold standard”) and drug susceptibility testing	Long TAT; lower yield in children/paucibacillary disease; requires trained staff, specialized infrastructure; higher biological risk; operationally complex
Automated NAAT	Sputum, induced sputum, gastric aspirate, tracheal aspirate, BAL, lymph node aspirate, tissue fragments, CSF, cavity fluids, urine, stool, blood/bone marrow	~90 min–2 h	Medium	BSL-2	Rapid TB detection and drug susceptibility testing (mainly rifampicin)	Cartridge/platform cost; limited drug-resistance detection
LAMP	Sputum, induced sputum, tracheal aspirate	~1 h	Low to medium	BSL-2	Low-complexity molecular alternative for rapid TB detection	Lower sensitivity than Xpert in HIV+/paucibacillary disease; mainly validated for respiratory samples; manual workflow; no resistance detection
LPA	Respiratory samples or culture isolates	~5–48 h	Medium to high	BSL-2/3	TB detection and antimicrobial resistance profile	Lower performance in paucibacillary samples (HIV+/children); targets only known mutations; Risk of contamination and subjective interpretation
LAM	Urine	~25–60 min	Low	BSL-2	Rapid test for TB in people living with HIV with advanced disease	Useful mainly in HIV-positive patients with low CD4; poor sensitivity in HIV-negative individuals and most extrapulmonary TB
TST	Intradermal PPD injection; reading of forearm induration	~48–72 h	Low	Not applicable	Screening and diagnosis of latent TB infection	Low specificity in BCG-vaccinated or NTM-exposed populations; reduced sensitivity in HIV+, immunosuppressed and young children; requires second visit; operator variability; not useful to diagnose active or extrapulmonary TB
IGRA	Peripheral blood	~1 day	High	BSL-2	Diagnosis of latent TB infection	Does not distinguish LTBI from active TB; variable sensitivity in HIV+ and young children; not indicated for extrapulmonary TB diagnosis; requires laboratory processing
mNGS	Sputum, induced sputum, BAL, CSF, tissue fragments, blood, cavity fluids, urine	~1–3 days	Very high	BSL-3	Pathogen detection in complex or extrapulmonary disease; identification of co-infections; exploratory resistance assessment	High cost and bioinformatics expertise; performance depends on pathogen load; limited availability in low-resource settings; longer and more complex workflows; centralized labs required
tNGS	Sputum, culture isolates, BAL, CSF, tissue, cavity fluids, stool	~1–10 days	Very high	BSL-3	Comprehensive genotypic drug-resistance profiling and surveillance after microbiological detection	Requires custom panels, specialized infrastructure and trained personnel; high cost; limited use in routine care; long TAT; performance reduced in very low-bacillary samples

Abbreviations: TAT = turnaround time; BAL = bronchoalveolar lavage; CSF = cerebrospinal fluid; TB = tuberculosis; HIV = human immunodeficiency virus; BSL = biosafety level; NAAT = nucleic acid amplification test; LAMP = loop-mediated isothermal amplification; LPA = line probe assay; LAM = lateral flow lipoarabinomannan assay; TST = tuberculin skin test; IGRA = interferon-gamma release assays; mNGS = metagenomic next-generation sequencing; tNGS = targeted next-generation sequencing.

## Data Availability

No new data were created or analyzed in this study.

## Abbreviations

The following abbreviations are used in this manuscript:

BCG	Bacillus Calmette -Guérin
BSL	Biosafety level
DST	Drug susceptibility testing
ELISA	Enzyme-linked immunoassay
HIV	Human immunodeficiency virus
IGRA	Interferon-gamma release assay
IFN- γ	Interferon-gamma
LAM	Lipoarabinomannan
LAMP	Loop-mediated isothermal amplification
LF-LAM	Lateral flow immunoassay
LPA	Line probe assay
LTBI	Latent tuberculosis infection
MDR	Multidrug resistance
MGIT	Mycobacteria Growth Indicator Tube
mNGS	Metagenomic next-generation sequencing
*M. tuberculosis*	*Mycobacterium tuberculosis*
NAAT	Automated nucleic acid amplification tests
NGS	Next-generation sequencing
PCR	Polymerase Chain Reaction
PPD	Purified protein derivative
RIF	Rifampicin
TAT	Turnaround time
TB	Tuberculosis
tNGS	Targeted next-generation sequencing
UV	Ultraviolet
XDR	Extensively drug-resistant
WHO	World health organization
